# The vulnerability to alcohol, tobacco, and drug use of adolescents in Hong Kong: a phenomenological study

**DOI:** 10.1186/s12887-019-1678-1

**Published:** 2019-09-02

**Authors:** Yim Wah Mak, Doris Leung, Alice Yuen Loke

**Affiliations:** School of Nursing, The Hong Kong Polytechnic University, Hung Hom, Kowloon, Hong Kong Special Administration Region, China

## Abstract

**Background:**

In Hong Kong, the use of alcohol, tobacco, and other drugs (ATOD) is associated with strong peer influences; frequently absent parents; academic pressures; and a lack of interpersonal skills to cope with stress and conflict. It is posited that this social context alters the nature of the adolescent risk of using ATOD. The study aimed to explore how social interactions in their local context shape experiences of adolescents who smoke or use alcohol with their parents and other significant people (e.g., teachers, peers) in their lives.

**Results:**

The participants consistently indicated that the communication of risk was fundamentally influenced by the attachment between the primary parent(s) and the child. In secure attachments, parents could positively discourage ATOD use by instilling fear or expressing regret or disappointment over its use. However, some parents expressed an overly permissive attitude about ATOD use, or stated that they had a limited ability to influence their child, or that the harm arising from their child’s use of ATOD would be minimal. Under these conditions, the authors posited that the potential influence of peers to disrupt parental attachments was stronger.

**Conclusions:**

Descriptive phenomenology was adopted in this study and Colaizzi’s method was used to analyse the collected data. Focus group interviews were conducted with 45 adolescents, 11 parents, and 22 school teachers and social workers in two districts in Hong Kong. A secure attachment between a parent and a child enhances the child’s sense of self-efficacy in avoiding addictive behaviours such as ATOD use. In contrast, insecure parent-child attachments may trigger children to resist social norms, and disrupt their parental attachments. In these instances, parents may inadvertently convey the message that their children do not need protection from the risks of using ATOD. The key findings suggest that reinforcing secure parental attachments, as well as emphasizing how messages of vulnerability to ATOD are conveyed, may counter balance pressures (including peer influence) to use these substances. Further research is needed to uncover mechanisms of communication that add to the vulnerability of adolescents to using ATOD, and to the negative long-term consequences from ATOD use.

## Background

Research into the origins of substance use problems increasingly point to early adolescence as a critical period – one predictive of later problems [1]. About one-third of adolescents begin drinking by age 13 and 10%, begin by age 10; while tobacco use may begin earlier [2]. Adolescents who smoke or use alcohol tend to demonstrate more problems with social and behavioural adjustment than those who do not [3], including exhibiting a long-term pattern of risky sexual behaviour, driving while intoxicated, and using other drugs [4].

Research in this area tends to focus on the use of alcohol, tobacco, and other drugs (ATOD) triggered by both positive and negative personal events (e.g., failing grades, celebrations) that cause anxiety. These events are commonly predicated on problematic social relationships, especially with parents suggesting that these relationships may either deter or trigger continuing ATOD use/abuse [5–7]; in addition to broader social structures, which marginalize groups. For example, American Indians/Alaskan native youth are at a particularly great risk of abusing various substances when compared to other minority groups in the United States [2]. This is theorized to be associated with the loss of a cultural base/community, and due to historical trauma [8].

Research concerning parental influence on the vulnerability of adolescents to using and abusing ATOD is mixed. On the one hand, Chan et al. (2013) reported that a study in Australia found that parental attachment was unrelated to trajectories of alcohol use in adolescents. Only the lack of parental supervision at grade 9 (age 15 years old) was associated with a ‘steep escalation’ in alcohol use [4]. On the other hand, Mathijssen et al. (2014) reported that, from the perspective of adolescents, parental influence ‘appears to play the most important role in the prevention of alcohol use,’ as strict rules appear to discourage adolescents from starting to drink early and progressively more (p. 872) [7].

Mixed results about parental influence appear dependent on the nature of the parent-child relationships [9–11]. More specifically, the attachment to mothers correlates more strongly to alcohol consumption than does attachment to fathers [7]. Further, numerous studies have suggested that when parents smoke, this increases the tendency of children to experiment with smoking [12]. As a result, the exposure by children to second-hand smoke at home from their parents appears to strongly increase the risk that adolescents will initiate smoking [12].

Bowlby (1988), a proponent of attachment theory, theorized that parental influences may essentially structure the vulnerability of children to tobacco and alcohol use and to other risks in their environment [6, 13, 14]. According to Bowlby (1988), parental patterns of behaviour establish the foundation of a secure attachment during a child’s early years of development [13, 14]. The individual’s sense of security in receiving protection from threats in the world regulates his/her expectations of others [13, 14]. The expectations of others become the individual’s ‘working models’ and signify ‘the worthiness of the self in relation to significant others, as well as the availability and responsiveness of attachment figures’ [15]. Hence, if parents are not attentive, or are dismissive or abusive, individuals may develop a sense of insecurity, that is, attachment anxiety and/or avoidance [15] and find it difficult to regulate their emotions when under stress [13, 14]. On the one hand, individuals will persistently seek reassurance from significant others, demonstrating anxious attachment. On the other hand, individuals may minimize their distress and become overly self-reliant, demonstrating attachment avoidance [15].

The importance of secure attachments was discovered in a longitudinal study conducted in the Netherlands by van der Vorst et al. (2006), ‘The lower the adolescent perceives the quality of the attachment relationship to be, the more likely the adolescent is to consume alcohol at an early age’ ([6], p., 113). For this study, the quality of the attachment relationship was defined by indicators of attachment security: ‘the combination of low anxiety and low avoidance, reflecting feeling comfortable with closeness and trusting’ that a parent ‘will be available and responsive when needed’ ([15], p., 500).

The theory of social control also may be used to explain why parental monitoring lowers alcohol use. This theory suggests that when one’s moral values become internalized, this may limit the desire to engage in deviant behaviours [7]. However, the theory does not take into account how one’s moral values or attitudes are essentially reinforced by social norms, including norms involving parental communication or influences of others (i.e., peers, teachers). Moreover, the current evidence suggests that for adolescents, the attitudes of their peers more prominently trigger A&T use than their parental relationships [7]. Van der Vorst et al. (2006) even suggested that early alcohol use has a negative influence on parental attachment [6]. In other words, ‘the more the young adolescent consumes alcohol, the less strong the adolescent perceives the attachment relationship with his or her parents to be’ (p. 114). Hence, alcohol use in adolescence may indicate that the child’s emotional attachment with his or her parents is weakening, adding to the controversy about how continued substance use and abuse is structured later in life.

In the Hong Kong Special Administrative Region (HK) of China, prosocial cultural norms (transmitting moral values) more explicitly discourage deviant behaviour [16, 19]. However, in a survey conducted in HK in 2005–2006 involving 884,300 children aged 14 and below, 2.2% of children aged 11 to 14 years were reported to have smoked; among those, 22.1% had begun doing so at the age of 10 or younger [17]. In comparison, 5.0% of children aged 11 to 14 years old reported that they had drunk alcohol, including beer; among those, slightly more than one-third had had their first drink of alcohol at the age of 10 or younger [17].

Shek (2007) pointed to several factors that may have increased the vulnerability of adolescents in HK to substance abuse: a) strong peer influences, including via access to virtual communities; b) frequently absent parents working across the border or due to increasingly more non-intact families; c) ‘a morbid emphasis on achievement’; and d) a lack of formal and informal interpersonal skills/training to cope with stress and conflict [18]. The combination of all these factors, with relatively little adversity in HK adolescents’ lives, are suggested to be barriers to developing resilience within a search for life meaning, and increase their vulnerability to negative interpersonal influences [18]. Further, Shek (2007) argued that, given the pessimism that young people have about their future social mobility, there is a growing tendency among adolescents in HK to ‘normalize’ and even justify the use of substances to cope [18].

In Hong Kong, the proximity (physical and emotional) of adolescents to their parents tends to extend into their early adulthood for longer than in Western cultures such as Australia and the United States [19]. In doing so, the working models of when they are expected to be self-reliant from their parents, may culturally differ from other parent-child relationships [20]. Thus, the emotional proximity of adolescents and their vulnerability to threats in their relationships, such as from ATOD use, are posited to operate beyond physical boundaries. However, the authors suggest they are inherent in the child’s sense of security, and temporal to social norms of where an individual grew up as a child, more than to where a person is living as an adolescent or young adult. To explore these ideas from a culturally specific context, the authors explored students’ perceptions in the distinct culture of Hong Kong.

Research on school-based substance abuse prevention programmes show that they are effective in helping to reduce the risk that adolescents will engage in smoking tobacco [21] and drinking alcohol [22], particularly during the developmental period from primary to secondary school [22]. Such programmes might potentially counter the effects of negative parenting. Most of the studies on such programmes have tended to focus on parents’ and children’s perceptions of these programmes [23]. However, a deeper and more detailed understanding of their experiences is desirable. In addition, the views of stakeholders who deliver the programmes, such as teachers and social workers, tend not to be represented [24, 25].

### Purpose

The aim of the study was to explore perceptions of parents, adolescents, teachers, and social workers, about what causes adolescents in HK, to use (or not use) ATOD. The research questions were: (1) How do social interactions (with his/her parents and other people of significance) shape experiences of ATOD in their local contexts? (2) What are persistent patterns shaping communication of adolescent use of ATOD, the risks of using ATOD, and the ways to prevent such use?

## Methods

### Setting of the study, recruitment and sampling

Adolescents, parents, social workers, and teachers in two districts of HK were recruited. Adolescents are considered most vulnerable to using substances when family incomes are low [26, 27]. Among 16 districts of HK, the two districts that were selected reported the lowest monthly household income in HK [28]. Principals of secondary schools in the districts were approached for permission to conduct interviews in their schools. Some school principals introduced members of the research team to the presidents or teachers of their Parent-Teacher Association to allow the research team to explain the study’s purpose and expectations. Parents were recruited through invitation by teachers or members of parent-teacher associations. Social workers were recruited from community centres located in the target districts. Adolescents that used A&T, or friends of students who smoked or drank, were included with the help of their teachers. Participants with direct knowledge of the use of A&T were recruited through purposive sampling. The criteria for inclusion were: primary or secondary school students, parents of children in primary or secondary school, and social workers and teachers in primary and secondary schools. Researchers took great care not to invite parents who had adolescents participating in the study. This was done to allow the parents to freely express their views, without worrying that they might violate the confidentiality of their children or that their adolescents would encounter negative consequences as a result of the parental disclosures.

The participants were given information explaining the study, and measures to assure confidentiality and anonymity were undertaken (i.e., data were securely stored, pseudonyms were used). Written informed consent was obtained from all of the participants, including teachers and social workers, before the commencement of data collection. After obtaining permission from the school principals, adolescent students were recruited through brief introductions delivered during lunch breaks, as well as posters. Passive parental consent for their participation was obtained by providing the students with an information sheet and a refusal form to bring home to their parents. Parents were asked to send back the refusal form to the research team if they did not want their child to participate. Even with parental consent obtained, student participation was also voluntary, and written assent was also obtained for participants less than 18 years of age. The Human Subjects Ethics Committee of the Hong Kong Polytechnic University approved the research design and this consent procedure for participants.

### Data collection

Focus group interviews were conducted with: a) students, b) parents, and c) school teachers and social workers. (Note: Social workers and teachers were grouped together.) Special arrangements were made for those who preferred individual interviews. The focus group interviews took place in either an activity room in the community centre or in interview rooms at the schools. Each student focus group was comprised of three to six participants of mixed gender. Those similar in age were grouped together. Social workers and teachers were classified as being in one group, as they shared a similar background and experiences in handling the developmental problems of adolescents. For the most part, the majority of parents participated in a focus group together. However, due to issues of availability, individual interviews with some parents were conducted. All focus groups were co-led by two researchers who had experience in moderating groups, at a time convenient to the participants (after regular school hours).

In the focus group interviews, the researchers used a semi-structured interview guide (see the list of open questions in Table 1) to ask questions concentrating on three areas: (1) knowledge and attitudes towards ATOD use; (2) parent-child communication on the use of ATOD, the risks of using ATOD, and how to prevent such use; and (3) perceptions of communication with significant others (e.g., parents, teacher, peers) that prevents or shapes ATOD use. All of the focus group and/or individual interviews took about an hour, and were audio-recorded and transcribed verbatim. Data collection, carried out by two members of the research team (MYW and a research assistant), continued until patterns began to repeat themselves [29].

**Table 1:** Interview guiding questions for children / adolescents, parents and teachers / school social workers

**Interview guiding questions for children / adolescents:**	
1. Do you think that the family has any way of preventing teenagers from smoking or drinking alcohol? Follow-up questions: a) How do you know about these precautions? b) Why would you choose these methods?	
2. Have you talked to your parents about smoking and drinking? Follow-up question: Could you please describe the situation at that time?	
3. Have your brothers / sisters or friends talked to their parents about smoking and drinking?	
4. What kind of method or type of communication do you think is feasible or does not work? Why?	
5. Do you encounter difficulties at times? How do you and your parents deal with these difficulties?	
**Interview guiding questions for parents:**	
1. As a parent, what can you do to prevent your child from smoking or drinking alcohol? Follow-up questions: a) How do you know about these precautions? b) Why would you choose these methods?	
2. Have you talked to your children about smoking, drinking, and drug use? Follow-up questions: a) Do you think that the approach to communication or type of communication that you use with your child is working or will work? Why? b) Is your child / Are your children in trouble and how are you dealing with the situation?	
**Interview guiding questions for teachers and school social workers:**	
1. Do you think that a parent can prevent his/her child from smoking or drinking? Follow-up questions: a) How do you know about these precautions? b) Why would you choose these methods?	
2. What do you think of how today’s children communicate with their parents?	
3. What do you think that parents talk to their children about when they deal with the issue of smoking, and drinking? Follow-up question: Do you think that the approach to communication or the type of communication between parent and child is working or will work? Why?	

### Data analysis

Data were analysed using descriptive phenomenology, in accordance with Colaizzi’s method [30] Descriptive phenomenology originated with Husserl (1960) to describe the ‘essential’ structure of a phenomenon [31, 32]. Analysis was performed using Chinese transcripts, which were then translated into English for a final analysis.

Credibility was established through prolonged engagement with the interview data, which were repeatedly reviewed by two researchers (YWM and a research assistant). First, the researchers independently read each transcript and identified relevant chunks they called ‘meaning units.’ Then, they met to discuss the patterns that they had found in the data and came to a consensus on the preliminary themes.

Once the preliminary findings were established, the participants were asked to provide feedback on these findings and about whether the meanings and concepts of the analysis were consistent with what they intended to express. Next, a third researcher (DL) joined the team to re-contextualize the relevant themes and sub-themes. The researchers (YWM, a research assistant, and DL) then repeatedly checked the data against the themes and sub-themes to determine their plausibility [29].

Finally, relevant quotations were translated into English for discussion amongst the three researchers. The authors endeavoured to provide a balanced mix of interpretations and rich descriptions of data, to be able to compare their interpretations to determine how they fit with broader conceptualizations of ATOD use, and to render the findings transferable to similar contexts with similar groups of people.

## Results

Focus group / individual interviews were conducted with 45 adolescents, 11 parents, and 22 school teachers and social workers in two districts in Hong Kong (See Table 2 summerised statistics on the participants).

**Table 2:** Sumerised statistics on the participants

Type of Participants	Number of Groups	Number of Participants
Students	9	45
Parents	5	11^a^
Teachers/Social Workers	4	12^b^

^a^For parent participants, five focus group interviews and one individual interview were conducted

^b^For teacher/social worker participants, four focus group interviews and one individual interview were conducted

### Social workers’/teachers’ group

A total of 12 social workers and teachers participated in either the group or individual interviews, depending upon their availability. Two of the 12 were social workers, with one working as an on-campus social worker and the other as an outreach social worker. The remaining 10 interviewees were junior and senior teachers and teaching assistants at the participating schools. One was a vice president and two were discipline-specific teachers. Three were lower-form class mistresses or masters, and one of the teaching assistants was a member of the school’s counselling team.

### Students’ group

A total of 45 students ranging from primary five to secondary six students were interviewed in groups. The interviewees were asked whether they had ever tried to smoke, drink, or take drugs. Their report of their previous experience with using alcohol, tobacco, and other drugs did not indicate that they were regular or frequent users.

Of the 45 students, two were primary school students and the rest were secondary school students. Seventeen of them (37.8%) reported using at least one type of ATOD. Paternal, maternal, and sibling ATOD use was reported by 77.8% (*n* = 35), 17.8% (*n* = 8), and 6.7% (n = 3) of the respondents, respectively. The majority of the participants perceived that A&T prevention was either important (*n* = 25, 55.6%) or very important (*n* = 9, 20%) (see Table 3 summerised characteristics of student participants and their experience using ATOD).

**Table 3:** summerised characteristics of the student participants and their experience using ATOD

Edu level	n	Self ATOD use	Father’s ATOD use	Mother’s ATOD use	Sibling’s ATOD use	ATOD prevention
Year4	4	None (*n* = 3);	A,T (*n* = 1); Smoke (*n* = 1); None (*n* = 1)	None (*n* = 4)	None (n = 3)	V (n = 1); X (n = 1); XX (n = 1)
Year 5–6	3	None (*n* = 2) A,T (n = 1)	A,T (n = 1); T (n = 1) A (n = 1)	None (n = 2) A (n = 1)	None (n = 2) No siblings (n = 1)	V (n = 3)
Year 7	7	A,T,OD (n = 2); A,T (n = 1); A (n = 1); None (n = 3)	A,T (n = 2); A (*n* = 2); T (n = 1); OD (n = 1); None (n = 1)	A,T (n = 1); A (n = 1); T (n = 1); None (n = 3)	ATOD (n = 1); A (n = 1); T (n = 1); None (n = 2) No siblings (n = 2)	V (n = 3); VV (n = 4)
Year 8	9	A,T (n = 1); A (n = 1); T (n = 1); None (*n* = 6)	A,T (n = 4); A (n = 1); T (n = 1); None (n = 2)	A,T (n = 1); None (n = 6);	A (n = 2); None (n = 3); No siblings (n = 1)	V (*n* = 5); VV (n = 2); X (n = 1); XX (n = 1)
Year 9	8	A,T (n = 1); None (*n* = 7)	A (n = 1); T (n = 3); None (*n* = 3);	A (n = 2); None (n = 5)	None (n = 7);	V (n = 5); VV (n = 1); X (n = 1); XX (n = 1)
Year 10	11	A,T (n = 3); T (n = 4); None (n = 4)	A,T (n = 2); A (n = 4); T(n = 2); OD (n = 1); None (n = 2)	A,T,O,D (n = 1); A,T (n = 1); None (n = 8);	None (n = 8); No siblings (n = 3)	V (n = 6); VV (n = 1); XX (n = 4)
Year 11	2	None (n = 2)	T (n = 1); None (n = 1)	None (n = 2)	No siblings (n = 2)	V (n = 1); VV (n = 1)

Abbreviations Index: F = Female; M = Male; A = Alcohol use; T = Tobacco use; OD = Other drug use; VV = Very important; V = Important; X = Not important; XX = Very unimportant; − = None

Note: The number may not add up to the number of participants due to missing data

### Parents’ group

A total of 11 parents participated in the focus group interviews. All of the participating parents had at least one child studying at a primary or secondary school in HK, but they were not the direct guardians of the students who were invited to participate in the study (See Table 4 characteristics of the parent participants).

**Table 4:** Characteristics of the parent participants

	Relationship with children	No. of children
P1	Mother	3
P2	Mother	1
P3	Mother	1
P4	Mother	2
P5	Mother	3
P6	Mother	2
P7	Mother	2
P8	Mother	2
P9	Father	1
P10	Mother	1
P11	Mother	/

Abbreviation Index: n/a = Not applicable; / = Not provided

### Primary themes

**Fig. 1:**
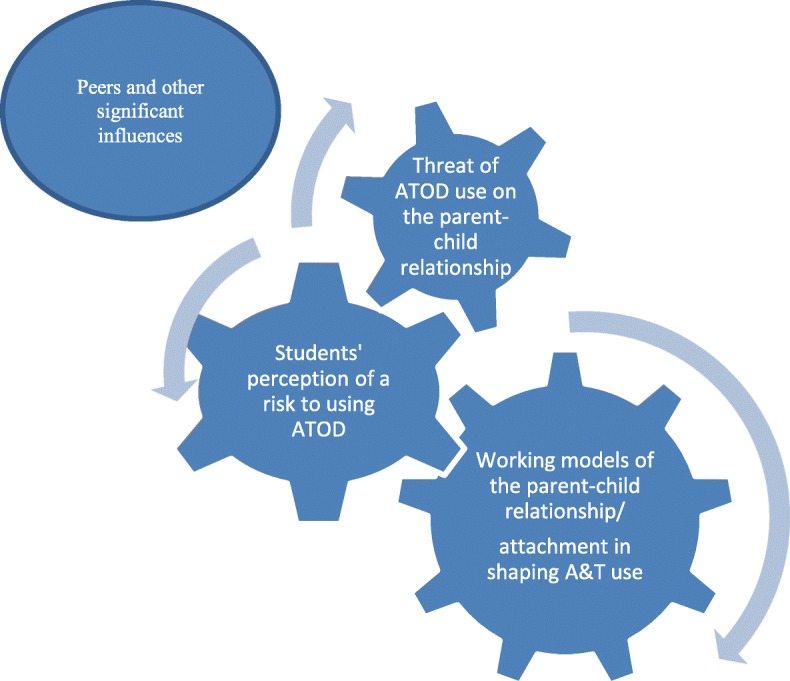
Primary Themes

The social nature of the adolescents’ experiences was based on three essential structures/ themes: (1) The working models of child-parental attachment in shaping adolescents’ A&T use; (2) Students’ perceptions of their vulnerability to ATOD use and the risks to parent-child attachments; and 3) Potential peer influence disruptive of parent-child attachments (see Fig. 1). Participant exemplars are identified by group designation, sex (M/F), and if available, their age (years). (S: Student, P: Parent, T: Teachers or social workers).

#### Theme 1: the working models of child-parental attachment in shaping adolescents’ a & T use

All of the informants identified structures that shape a child’s expectations of his/her parents, as playing a critical role in how they saw themselves using ATOD. More specifically, the issue was whether the nature of the parental relationship was one that conveyed a tone of care or worthiness of concern from parents. If students talked of ‘caring’ or being ‘loved’ by their parents, this meant that they occupied a position of concern in their family, which could be threatened by the use of ATOD. One student expressed the view that ‘most parents’ might not ‘have the time’ to express words of concern or care that their children are at risk of using ATOD:

*S34, M, 16 yrs: I think parents can prevent their children from A&T use by educating them. However, most parents do not have much time to do that. So I think that the parents’ expression of caring for their children in their communication and interaction is valuable. Children who feel the love will not try A&T so as not to disappoint their parents.*

Most adolescents stated that their parents expressed a need to protect them from the risks of ATOD use by warning them to ‘stay away’ from substances because of the potential negative consequences. This appeared to imply that parents had created a working model of what would happen if adolescents used ATOD, such as the infliction of corporeal punishment or the destruction of their relationship:

*S14, M, 15 yrs: My family instructed me not to take drugs; otherwise they will beat me to death. That’s why I don’t want to take drugs.*

*S19, F, 10 yrs: I think telling children about the negative consequences of smoking, including that it is [illegal], can prevent them from smoking.*

*S31, M, 13 yrs: My parents told me that drug use can cause death [absolutely no tolerance for the child’s ATOD use].*

While these examples did not necessarily reflect ‘positive’ parenting, they conveyed a strong stance that adolescents perceived that they would be vulnerable to the negative consequences of using ATOD, when and if their parent-child attachments were ones that conveyed emotional proximity, and perhaps also depending on the parents’ capacity to respond to their children.

In other words, adolescents’ perceptions of parental warnings were powerful in triggering feelings of guilt in the adolescents when, and if, they considered using ATOD. Some students expressed a wish to cut down on their consumption of ATOD out of consideration for their parents:

*S12, M, 15 yrs: I smoke less knowing that my parents are unhappy about my smoking and blame themselves for not teaching me properly. And I will try very hard to reduce my smoking.*

All of the teachers expressed strong opinions on how parents ought to convey a moral stance that could threaten their relationship. They expressed a need for parents to act as role models and demonstrate expectations for their adolescents on whether ATOD use was socially acceptable or not. In particular, teachers reported that by not consuming ATOD, parents took an important stance against their use:

*T8, F, 40 yrs*: *If parents can provide their children with good examples of living a positive and ATOD-free lifestyle, their children will adopt the same values and follow suit. If children lack a positive role model from their parents, they may follow the same behaviours and lifestyle of their parents.*

In comparison, whether or not parents actually used ATOD was not as important as setting up working models as to their acceptability within the parent-child relationship. This was exemplified in one parent’s narrative indicating that the use of ATOD was not discouraged during his/her own childhood. Other parents reported that it was important to express an attitude of ‘not being like me’ to their children:

*P8, F, 35 yrs: I started smoking when I was a kid. I advised my children not to smoke like me, and I know that second-hand smoke is not good for them either. My children told me that they know that smoking is not good for them, and I praised them for not trying it.*

Some students expressed the view that their parents’ lack of concern, or lack of respect towards them, generated mistrust and estrangement, which has been theorized as being a form of attachment avoidance. They particularly resented their parents comparing them to other ‘good kids’, and disclosing their personal or private matters to others. Other students said that their parents were too strict, or that their parents acted in ways that were ‘annoying’:

*S22, F, 10 yrs: When I talk to my parents, they will start praising other children who score highly or get a passing mark.*

*S32, M, 12 yrs: My parents are super annoying! They keep asking me not to do this and not to do that. I don’t want to listen to them.*

The authors posited that these working models of their parents’ relationships and expectations shaped the nature and structure of the students’ perception of risk, prompting them to not only experiment but, perhaps more importantly, disrupt their parental attachments with the continued use of ATOD. This is revealed in theme 2.

#### Theme 2: students’ perceptions of their vulnerability to ATOD use and the risks to parent-child attachments

All of the students’ expressed some knowledge of the potential negative impacts of ATOD use, and tried to convey the message that they would limit their use of these substances. However, as the findings will reveal, their perceptions of the risks of ATOD did not vary, as much as their *perceptions of their vulnerability* to (and hence expectations to be protected from) the risks of ATOD. These perceptions of vulnerability did not necessarily have an impact on whether or not the children experimented with ATOD, but might affect their patterns of continued use (and perhaps, future abuse), thus disrupting or sustaining their parents’ working models of what was acceptable (expectations).

Communication about ATOD was influential when students conveyed the view that they held permissive attitudes toward the use of ATOD, and parents appeared to minimize their children’s need to be protected from using ATOD, or delegated to others the responsibility of providing such protection.

- ***Students’ permissive or ‘open’ attitudes towards A&T aligned with working models (expectations) of parents***

When students were asked about what they thought of ATOD use, quite a few revealed that their parents permitted them to smoke and drink. Parental knowledge (or lack thereof) may have inadvertently minimized their children’s need to be protected from using ATOD (perhaps to avoid conflicts and preserve harmony with their children). Hence, adolescents’ *perceptions of their vulnerability* to the risks was low. Further, some student participants told us that they smoked or drank with their parents, or that their parents supplied them with alcohol and cigarettes:

*S16, M, 15 yrs: My father gave me permission to drink his glass of alcohol, so I drank.*

*S6, M, 15 yrs: I smoked together with my mom in the kitchen.... She even gave me $50 to buy a package of cigarettes for her.*

As this excerpt reveals, the expectations of the parents were reinforced by working models indicating that students could use ATOD and that this would not threaten their relationship with their parents.

b) ***Parents’ belief that their adolescents do not need to be protected from the risks of ATOD use, or their delegation to others of the responsibility of providing such protection***

Some parents felt that their adolescents did not (yet) warrant protection from the risks of ATOD use because they were still too young to be vulnerable to such risks or had not demonstrated such vulnerability:

*P9, M,*: *My children are still young, and too young to talk about ATOD use.*

*P4, F: My child has a ‘good’ personality and is innocent, [so] he won’t take up ATOD.*

In other instances, a few parents expressed a desire to delegate this responsibility to others, including the adolescents themselves, for various reasons. One parent stated that she lacked communication skills and/or did not ‘know how’ to talk to her adolescents about using ATOD. Thus, this parent felt that she had a limited capacity to impact her/his adolescents’ use of ATOD:

*P4, F,32 yrs: I have never thought of how to prevent my children from using ATOD …*. I *cannot think of any ways to prevent children from using ATOD.*

A number of teachers expressed the belief that lower-income parents and parents who worked long hours generally lacked the energy or time to convey knowledge, and that this demonstrated a lack of adequate concern, which shaped the use of ATOD:

*T3, M, 50 yrs Parents prefer to spend their non-working hours resting to spending time with their children, which gradually leads them to lose control over their children when the children enter secondary school.*

Some teachers expressed the belief that such parents had shifted the responsibility of protecting adolescents from the risks of using ATOD to schools:

*T9, F, 28 yrs: Some parents place the responsibility of educating their children entirely on the school. The father of one of my students told me that he didn’t have a wife and he didn’t know how to teach his son. He sees it as the school’s job to put his son on the right track.*

One parent (P3) minimized her own influence and that of other parents on shaping their children’s views as they grew up, suggesting that the decision on whether or not to use ATOD was their adolescents’ to make when they grew older:

*P3, F, 40 yrs: When children have grown up, they have their own view. Whether they will take up ATOD depends on how much they can discipline themselves.*

### Theme 3: potential peer influence disruptive of parent-child attachments

Some students agreed that their parents were not capable of preventing them from using ATOD, not because they lacked influence on them, but because parents did not pay enough attention to their adolescents or spend enough time with them. This kind of parental communication appeared to convey a lack of concern for their adolescents’ vulnerability, and/or to signal a dependence on the adolescents themselves to deal with the problem when it arose. In some instances, students admitted to talking to their peers about problems, rather than their parents:

*S30, M, 13 yrs: My parents don’t talk to me; they are too busy to realize that I feel sad.*

*S2, F, 14 yrs: I talk to my friends when I have decisions to make. I never talk to my parents about my problems.*

Once more, the students’ inability to perceive that there are risks to using ATOD, and what those risks are, further confirmed to the authors that those students demonstrated a kind of attachment avoidance, and may have been overly self-reliant when it came to the decision on whether or not to use ATODs.

On the other hand, if they had a form of anxious attachment, some students might turn to their peers to support decisions about the risks of using ATOD. Some expressed permissive attitudes towards A&T, stating that they ‘liked the taste of alcohol and cigarettes’, and that consuming these substances was a means of ‘releasing pressure and enjoying life’. All in all, these students found the use of ATOD to be socially acceptable, and some had family that reinforced these messages:

*S29, M, 15 yrs: We (with friends) talked and heard a lot about ATOD use. One might find it astonishing when one hears of it for the first time. But as I heard and saw more, it become very common to me and no big deal. I was surprised when I saw my friend taking drugs for the first time. But after several times, it is really not a big deal. I am not taking drugs now, but I am not sure if I would not in the future.*

In these instances, if the working models (attachments) with parents demonstrated anxious attachments or attachment avoidance, the authors posited that students were more likely to value peer influences, and perhaps be more likely to be vulnerable to disrupting the parent-child attachment, through experimentation with ATOD and the continued use of those substances.

Some participants identified peer influence as the main reason for initiating cigarette smoking, and for why they continued to smoke or did not resist the temptation to smoke when their peers smoked. Indeed, teachers concurred that the knowledge that their peers were using ATOD motivated adolescents to try ATOD. Some teachers perceived that peer groups could exert a strong influence on disrupting family norms (i.e., parents did not drink):

*T3, M, 50 yrs: A student studying in year 1 bought a couple of cans of beer and drank them at school. He got drunk. His parents were notified and were surprised about his drinking, and told us that both parents do not drink at all. The student told us that he witnessed a classmate buy a can of beer that morning, and wanted to try it. As you see, although school and family play an important role in shaping children’s conduct, sometimes peer groups have [the] strongest influence.*

While the authors agree that a need to belong to their peer group might put all children at a risk of using ATOD, we theorize that this risk is dependent on the strength of the family connection and family norms. First and foremost, it depends on whether the students value their parent-child attachments enough to prevent them from using ATOD, or whether they would risk disrupting this attachment through the use and abuse of ATOD with peers.

To illustrate our theoretical model, the authors suggest that the temporal and evolving nature of parent-child attachments in the child’s developmental trajectory may be threatened by the child’s perception that there is any threat to himself/herself and to the parental relationship, such as from using ATOD. Furthermore, peers may contribute to a disruption in the parent-child attachment, when the parent-child relationship is weak, placing children at a greater risk of using ATOD to cope.

## Discussion

In our study, the nature of the adolescents’ experiences with ATOD appeared to be rooted in their attachment to their parents (a degree of secure or insecure attachment), which was based on their perceptions of the care and concern shown by their parents; as well as in how such an attachment influenced the adolescents’ perceptions of their vulnerability to the risks of using ATOD. This vulnerability was structured by the individual’s working models of his/her parental attachments. Further, this shaped the degree to which peers might threaten to disrupt family norms at any moment in their developmental trajectory.

Positive parental caregiving, which acknowledges the vulnerability of adolescents and their need for protection from addiction, breaking the law, or risking death due to ATOD use, was particularly potent in parental language that discouraged adolescents from using ATOD. In contrast, behaviour conveying the perception that adolescents did not warrant protection from the risks of ATOD, or the delegation of this responsibility to others (perhaps to avoid conflict with their children), may have inadvertently minimized adolescents’ perception of their vulnerability and their need for parental concern regarding this risk. Drawing on Bowlby (1988), the authors theorize that these perceptions of vulnerability trigger a belief in individuals that they are valued and cared about [17], but may not necessarily have an impact on whether or not adolescents experiment with ATOD. Rather, the authors posit that such perceptions of vulnerability may essentially structure patterns of use (and perhaps, future abuse) to cope, particularly if peers are knowingly (or not) used to disrupt family norms. In other words, the use of ATOD may be a way for adolescents to purposively resist or disrupt family norms, and separate from ‘annoying’ parents.

Consistent with other studies [33–35], our findings supported Bowlby’s (1988) theory that a child with a strong ‘attachment’ to his/her parents was perceived to be a concern, and warranted protection from ATOD use. As such, these children would be less likely to initiate consumption of ATOD, and/or be less likely to continue to use ATOD. Risk communication, as measured by adolescents’ perception of parental disapproval of the use of ATOD [36] was similarly found in our study to be a protective factor, influencing adolescents to avoid ATOD or reduce their use of those substances**.** Furthermore, the students in our study who found the effects of using ATOD to be pleasurable with peers, had difficulty stopping their use of ATOD. This was similar to the findings of another study [37], which reported that this might occur when the strength of peer influence disrupts the influence of parental attachment.

In contrast, the findings from our study suggest that parental attitudes may influence whether their children experiment with ATOD and, more importantly, whether their children may be at risk of continuing to use ATOD, if parents do not take the time or energy to discuss those risks. We refer to instances when parents themselves may use ATOD or express an ‘inability to control their children’; or when the parents appear to be inadvertently minimizing the risks of using ATOD (i.e., perhaps to delay having the conversation because they do not know how to discuss the subject). Hence, our findings support those of a previous study on the use of social control theory [7], which suggested that when adolescents receive the message that they do not need to be protected from these substances, or when parents delegate the responsibility for such protection to others, the adolescents may become resistant to prosocial norms, which hinder them from using ATOD.

However, unlike previous studies, our findings suggest that parents who nurture the development of prosocial norms in their adolescents based on a foundation of warmth and concern for their value, may discourage their adolescents from using ATOD. Moreover, adolescents who form secure attachments with their parents (low anxiety and low avoidance) may be more likely to express a willingness to stop using ATOD, so as not to disappoint their parents, as some participants in our study stated. This may be particularly relevant to cultures similar to that of Hong Kong.

In a study involving adolescents from four cultures in four countries, that is, the USA, China, Korea, and the Czech Republic, Dmitrieva et al. (2004) found that family factors of whether adolescents perceived ‘lower levels of parental involvement and higher rates of parent-adolescent conflict affected their parent-adolescent relationships, and in turn was related to higher levels of adolescent depressed mood’ ([38], p., 441). Similarly, the authors posit that parental working models in HK stem from parent-adolescent attachments created by higher relative opportunities for parental involvement throughout adolescence, *as well as* higher possibilities for conflicts to occur, triggered by a tendency to succumb to the pressure to be successful both academically and financially [11]. Indeed, parents, especially mothers, transmit to adolescents some idea of when they are expected to become independent and, in doing so, the adolescents’ own values are formed [20]. In HK, the desire to be self-reliant coexists with the expectation to also remain connected and obey parental social norms of control [20]. The authors theorize that this may exacerbate conflicts for developing adolescents in HK, which may only be resolved through re-enacting the attachment styles developed as a child. As such, attachment styles may predict whether and how adolescents will cope using self-comforting measures (e.g., using ATOD) or seek out family members or partners, if accessible [18].

To date, studies on how parental influences shape the social nature of adolescents’ patterns of behaviour and vulnerability to using ATOD have tended not to consider the temporal and evolving nature of the parent-child relationship nor social norms in the context of ATOD use. Our study indicates that the essential structure of parental attachment, in combination with parental knowledge, and continued involvement by parents with their adolescents, may explain differences in the continued use and possible risks of abuse of ATOD by adolescents. In addition, the local context of attitudes towards A&T appears to influence the propensity of a child/adolescent to be vulnerable to using ATOD.

### Strengths and limitations

One limitation of this study is that the findings were based on a particular sample of Chinese participants, mainly those recruited from two schools in districts in HK where the residents are of a relatively low socio-economic status. Therefore, the results may not be transferable to other ethnic groups and adolescent populations. Nevertheless, the use of a qualitative methodology in this study allowed for an analysis in which a theory of ATOD use was put forward from the perspective of various stakeholders and took into account local social norms.

## Conclusions

The key findings suggest that reinforcing secure parental attachments, as well as emphasizing how adolescents may be vulnerable to the risks of using ATOD due to the attitudes and actions of their parents and others, may counterbalance the pressures (including peer influence) that adolescents are under to use ATOD. The clinical implications of this study include providing training and support to parents on how to cultivate trust in parent-child relationships, helping parents to be good role models, to develop expertise on the risks of ATOD use, and to resolve conflicts in communicating about the need to protect their children from the risks of using ATOD.

Taking into consideration the nature of a child’s attachment to his/her parents draws attention to the specific relational needs of adolescents in a developmental trajectory known for its complexity. We suggest that a future study be conducted to examine the effects of attachment style on the vulnerability of adolescents to the perceived risks of using ATOD, based on their attachment style, and linked to their personal situations (e.g., social norms, parental support, peer influences). Further research is needed to uncover mechanisms of communication that add to the vulnerability of adolescents and the negative long-term consequences of ATOD use.

## Data Availability

The data supporting the findings are contained within the manuscirpt. The anonymised datasets used and/or analysed during the current study are available from the corresponding author Dr. Yim-wah Mak upon on reasonable request.

## Abbreviations

A&T: Alcohol and tobacco
ATOD: Alcohol, tobacco, and other drugs
HK: Hong Kong Special Administration Region of China
P: Parents
S: Students
SES: Socioeconomic status
T: Teachers

## References

[1] Hawkins JD, Catalano RF, Miller JY (1992). Risk and protective factors for alcohol and other drug problems in adolescence and early adulthood: implications for substance abuse prevention. Psychol Bull.

[2] Whitesell N, Asidigian N, Kaufman C, Big Crow C, Shangreau C, Keane E (2014). Trajectories of substance abuse among young American adolescents: patterns and predictors. J Youth Adolescence.

[3] Maggs J, Patrick M, Feinstein L (2008). Children and adolescent predictors of alcohol use and problems in adolescents and adulthood in the National Child Development Study. Society of the Study of Addiction.

[4] Chan G, Kelly A, Toumbourou J, Hemphill S, Young R, Haynes M (2013). Predicting steep escalations in alcohol use over the teenage years: age-related variations in key social influences. Addiction..

[5] Kostelecky KL (2005). Parental attachment, academic achievement, life events and their relationship to alcohol and drug use during adolescents. J Adolescence.

[6] van der Vorst H, Engles R, Meeus W, Dekovic M, Vermulst A (2006). Parental attachment, parental control, and early development of alcohol use: a longitudinal study. Psychol Addict Behav.

[7] Mathijssen J, Janssen M, Bon-Martens M, van Oers H, de Boer E, Garretsen H (2014). Alcohol-segment specific associations between the quality of the parent-child relationship and adolescent alcohol use. BMC Public Health.

[8] Dickerson DL, Brown RA, Johnson CL, Schweigman K, D’Amico EJ (2016). Integrating motivational interviewing and traditional practices to address alcohol and drug use among urban American Indian/Alaska Native youth. J Subst Abuse Treat.

[9] Parsai M, Voisine S, Marsiglia FF, Kulis S, Nieri T (2008). The protective and risk effects of parents and peers on substance use, attitudes, and behaviors of Mexican and Mexican American female and male adolescents. Youth Soc.

[10] Abar C, Abar B, Turrisi R (2009). The impact of parental modeling and permissibility on alcohol use and experienced negative drinking consequences in college. Addict Behav.

[11] Lam WK, Cance JD, Eke AN, Fishbein DH, Hawkins SR, Williams JC (2007). Children of African-American mothers who use crack cocaine: parenting influences on youth substance use. J Pediatr Psychol.

[12] Gilman SE, Rende R, Boergers J, Abrams D, Buka SL, Clark KA (2009). Parental smoking and adolescent smoking initiation: an intergenerational perspective on tobacco control. Pediatr..

[13] Flores PJ (2010). Group psychotherapy and neuro-plasticity: an attachment theory perspective. Int J Psychother.

[14] Bowlby JA (1988). secure base. Basic Books.

[15] Pietromonaco P, Uchino B, Dunkel Schetter C, Kazak AE, Klein W, Rothman A, Cameron L (2013). Close relationship processes and health: implications of attachment theory for health and disease. Health Psychol.

[16] Sun CF, Shek D (2010). Life satisfaction, positive youth development, and problem behavior among Chinese adolescents in Hong Kong. Soc Indic Res.

[17] Department of Health. Child Health Survey 2005-2006. Department of Health, Hong Kong. 2010. http://www.chp.gov.hk/files/pdf/chs_eng.pdf. Accessed 3 Aug 2019.

[18] Shek D (2007). Tackling adolescent substance abuse in Hong Kong: where we should and should not go. Sci World J.

[19] Rosenthal D, Feldman S (1991). The influence of perceived family and personal factors on self-reported school performance of Chinese and Western high school students. J Res Adolesc.

[20] Stewart S, Bond M, Deeds O, Chung S (1999). Intergenerational patterns of values and autonomy expectations in cultures of relatedness and separateness. J Cross-Cult Psychol.

[21] Thomas RE, McLellan J, Perera R (2013). School-based programmes for preventing smoking. Evid Based Child Health.

[22] Caria MP, Faggiano F, Bellocco R, Galanti MR, EU-Dap Study Group (2013). Effects of a school-based prevention program on European adolescents’ patterns of alcohol use. J Adolescent Health.

[23] Van Ryzin MJ, Fosco GM, Dishion TJ (2012). Family and peer predictors of substance use from early adolescence to early adulthood: an 11-year prospective analysis. Addict Behav.

[24] Peterson J (2010). A qualitative comparison of parent and adolescent views regarding substance use. J Sch Nurs.

[25] Feil EG, Severson HH, Walker HM (1995). Identification of critical factors in the assessment of pre-school behaviour problems. Educ Treat Child.

[26] Hill LG, Lochman JE, Coie JD, Greenberg MT, The Conduct Problems Prevention Research Group (2004). Effectiveness of early screening for externalizing problems: issues of screening accuracy and utility. J Consul Clin Psych.

[27] Finkelstein DM, Kubzansky LD, Goodman E (2006). Social status, stress and adolescent smoking. J Adolescent Health..

[28] Census and Statistics Department (2014). Population and Household Statistics analysed by district council district 2013.

[29] Spezaile HJ, Carpenter DR (2003). Qualitative research in nursing: advancing the humanistic imperative.

[30] Colaizzi PF, Valle R, King M, King M (1998). Psychological research as the phenomenologist view it. Existential phenomenological alternatives for psychology.

[31] Sanders C (2003). Application of Colaizzi's method: interpretation of an auditable decision trail by a novice researcher. Contemp Nurse.

[32] Husserl E (1960). Cartesian meditations: an introduction to phenomenology.

[33] Bernard HR (1988). Research methods in cultural anthropology.

[34] Chen CY, Storr CL, Anthony JC (2005). Influences of parenting practices on the risk of having a chance to try cannabis. Pediatrics..

[35] Cox RB, Roblyer MZ, Merten MJ, Shreffler KM, Schwerdtfeger KL (2013). Do parent–child acculturation gaps affect early adolescent Latino alcohol use? A study of the probability and extent of use. Subst Abuse Treat Pr.

[36] Looze M, Van den Eijnden R, Verdurmen J, Vermeulen-Smit E, Schulten I, Vollebergh W (2012). Parenting practices and adolescent risk behavior: rules on smoking and drinking also predict cannabis use and early sexual debut. Prev Sci.

[37] Henden E, Melberg HO, Rogeberg OJ (2013). Addiction: choice or compulsion?. Front Psychol.

[38] Dmitrieva J, Chen C, Greenberger E, Gil-Rivas V (2004). Family relationships and adolescent psychosocial outcomes: converging findings from eastern and Western cultures. J Res Adolesc.

